# Supervised exercise as adjunctive treatment for substance use disorder: Systematic review and meta-analysis

**DOI:** 10.1097/MD.0000000000045370

**Published:** 2025-12-26

**Authors:** Anas R. Alserihi, Abdulmajid M. Abdullah, Bader Bashrahil, Asim M. Albishry, Khalid W. Alansari, Safwan N. Khan, Ahmed Binmahfooz, Ahmad Alsaleh, Majed A. Alharbi, Moayyad Alsalem

**Affiliations:** a College of Medicine, King Saud bin Abdulaziz University for Health Sciences, Jeddah, Saudi Arabia; b King Abdullah International Medical Research Center, Jeddah, Saudi Arabia; c Psychiatry Section, Department of Medicine, King Abdulaziz Medical City, Ministry of the National Guard-Health Affairs, Jeddah, Saudi Arabia; d King Abdullah International Medical Research Center, Riyadh, Saudi Arabia; e King Department of Adult Mental Health, King Abdulaziz Medical City, Ministry of the National Guard-Health Affairs, Riyadh, Saudi Arabia.

**Keywords:** exercise, meta-analysis, mind-body exercise, substance, substance use disorder, SUD, systematic review, yoga

## Abstract

**Background:**

Substance use disorder (SUD) comprises a spectrum of cognitive, behavioral, and physiological symptoms, indicating that individuals continue to use substances despite significant substance-related problems. Recent evidence suggests multiple types of exercise as adjunctive or main intervention options for SUD. The study aimed to evaluate the effectiveness of supervised exercise in improving quality of life, mental health outcomes, and substance use in individuals with SUD.

**Methods:**

Medline, Cochrane Central Register of Controlled Trials (CENTRAL), and ClinicalTrials. gov were systematically searched for relevant randomized controlled trials. We assessed depression, anxiety, urine drug screen (UDS) and quality of life (QoL) of participants in the included studies. The revised Cochrane risk-of-bias tool assessed the quality of the studies. Regarding meta-analysis, effect sizes reported using standardized mean difference (SMD) and risk ratio (RR), the random-effects model, and the inverse variance (IV) technique. Grading of Recommendations Assessment, Development, and Evaluation (GRADE) criteria were used to evaluate the certainty of the evidence.

**Results:**

Eight studies were included in the qualitative review, whereas 4 were included in the quantitative synthesis. Exercise interventions demonstrated superior effects compared with standard treatment in improving outcomes for individuals with SUD in terms of QoL in the subdomains of physiology (SMD = 0.94, 95% confidence interval [CI]: 0.67–1.22, *P* < .00001, I^2^ = 0%), psychology (SMD = 0.89, 95% CI: 0.62–1.16, *P* < .00001, I^2^ = 0%), and social (SMD = 0.73, 95% CI: 0.31–1.14, *P* < .0006, I^2^ = 58%). However, exercise showed a similar effect to the control in the number of patients with positive urine drug screen (risk ratio = 0.79, 95% CI: 0.56–1.12, *P* = .18, I^2^ = 0%).

**Conclusion:**

Adjunctive exercise effectively improved the QoL of patients with SUD. However, it showed no effect on decreasing use. More evidence is needed to establish this as an adjunct to the initial lines of SUD interventions.

## 1. Introduction

Substance use disorder (SUD) is a complex chronic condition in which people who are addicted to alcohol, tobacco, or illicit drugs are unable to control their use despite their harmful effects.^[1]^ According to Substance Abuse and Mental Health Services Administration, 21% (61.2 million) of the US population aged 12 years or older have used illicit drugs in the past. Additionally, 16% (46 million) of the general population in the US, aged 12 or older, met the DSM-5 criteria for substance use disorder.^[2]^ The long-term use of addictive substances has a lasting consequence on changing brain structure, which is like changes present in other mental disorders such as mood disorders, schizophrenia, anxiety disorders, and impulse-control disorders.^[3]^ Addiction, as a chronic relapsing disorder, can significantly affect the quality of life in terms of financial, marital, physical health, and emotional aspects. Examples include impotence, decreased libido, aggressiveness, involvement in physical altercations, social isolation, and poor work performance.^[4]^ Nowadays, drug replacement therapy is one of the most used practices for the treatment of addiction. For instance, long-acting opioid agonists, such as methadone or buprenorphine, reduce the risk of relapse to the offending drug by decreasing powerful cravings and withdrawal symptoms and blocking the effects of heroin and other opioids. However, similar to all opioids, these medications are also prone to addiction and drug–drug interactions. One of the significant drawbacks of these drugs is the development of lower tolerance to the addictive substance during relapse. A relatively small amount leads to an overdose.^[5]^ This leads to stronger incentives and increased interest in finding an alternative treatment to the current modality of SUD. Exercise, in comparison to the standard treatment of opioid addiction, has shown that physical activities such as aerobics or endurance exercises (brisk walks, cycling, or swimming) and mind-body or balance exercises (tai chi and yoga) as an adjunct treatment have potential in the treatment of SUD.^[6,7]^ Physical activity combined with medical therapy for managing SUD has demonstrated an improvement in mood, mental well-being, and reduction in drug craving when compared to the group who were managed with medical therapy alone to treat SUD.^[8]^ On the contrary, other studies suggest that there is no convincing evidence on the role of physical activity in treating SUD. One study reported that aerobic physical activity for 3 weeks has not been shown to decrease alcohol abstinence rates.^[9]^ Hence, there is a need to analyze the effects of physical activity in SUD. This article conducts an extensive systematic review and meta-analysis of randomized controlled trials (RCTs) to assess the effectiveness of physical exercise as adjunctive therapy for substance use disorder compared to standard treatment; moreover, the focus is on depression, anxiety, urine drug screening (UDS), and quality of life (QoL).

## 2. Methods

This study used the Systematic Reviews and Meta-Analysis (PRISMA) checklist. Before the preliminary search, a protocol was developed and registered with PROSPERO (CRD42023381089).

### 2.1. Search strategy

The systematic search was conducted using Medline, Cochrane Central Register of Controlled Trials (CENTRAL), and ClinicalTrial.gov. The search strategy for this research is provided in File S1, Supplemental Digital Content, https://links.lww.com/MD/Q987. The search was not limited to specific dates or languages. The last search was done on January 23, 2023.

### 2.2. Study selection and data extraction

Six authors were divided into 3 pairs (Anas Alserihi and BB, Abdulmajid Abdullah and SK, KA and Asim Albishry). Each author independently screened the titles and abstracts of the reports; subsequently, the full reports were assessed for eligibility. Studies were included in the review if they met the following criteria: Adults diagnosed with substance use disorder by the DSM IV/V. Exercise intervention of any duration and intensity under supervision. RCTs. Studies measured the following domains: QoL, anxiety, depression, and craving. The exclusion criteria were as follows: Studies that included participants with other psychiatric diagnoses. Studies that included nicotine abuse. Disagreements within pairs were resolved using expert opinion (MA). The trial characteristics and results were extracted into a spreadsheet for data synthesis. Studies for which sufficient data could not be obtained were excluded from the quantitative synthesis and were instead included in the qualitative synthesis.

### 2.3. Statistical analysis and quality assessment

The meta-analysis was conducted using RevMan (Review Manager) version 5.4 (Cochrane Collaboration). The random effects model and inverse variance (IV) technique were used. The statistical significance threshold was a *P*-value <.05, with a 95% confidence level. I^2^ was used to assess statistical heterogeneity. The risk ratio (RR) was the effect size for dichotomous outcomes, whereas continuous outcomes were measured using standardized mean difference (SMD). In cases of multiple outcome observations, the latest follow-up visit was pooled in the meta-analysis. The Grading of Recommendations Assessment, Development, and Evaluation (GRADE) tool was used to judge the certainty of the evidence of each pooled estimate by assessing study design, number of included studies, risk of bias, inconsistency, indirectness, and imprecision. According to the latest recommendations, funnel plots were not assessed for publication bias because of insufficient studies in the data synthesis. The included studies have been organized into a table using the PICO format to assess their eligibility for both qualitative and quantitative syntheses. Following data extraction, Table 1 was created to present the information.

**Table 1:** Characteristics of the included trials.

Author	Country	Year	Number of participants	Gender		Mean age	Setting	Substance of addiction	Exercise				Control/comparison	Outcomes	Quality rating
				Male	Female				Type	Frequency	Duration	Intensity			
Sari et al^[10]^	Denmark	2019	175 (58 dropped from the study: 117 remaining)	84	33	44.6 (11.6)	out-patient	Alcohol	walking or running	twice a week for one-hour	6 mo	intensity not specified	standard treatment	1. Quality of Life: EQ-5D and EQ-VAS 2. Addiction Severity Index (ASI)	High Risk
Vedamurthachar et al^[11]^	India	2006	60	60	0	18–55 (36.5)	in-patient	Alcohol	Sudarshana Kriya Yoga	Once a day	2 wk	intensity not specified	continued inpatient care	1. Beck Depression Inventory (BDI) 2. Moring plasma cortisol 3. ACTH 4. Prolactin	Some Concerns
Li et al^[12]^	China	2002	86	86	0	Qigong group: 33.3 + 6.5, Medicine group: 31.9 + 5.9, Control group: 31.7 + 6.1	in-patient	heroin	qigong	2 to 2.5 h per day	10 d	intensity not specified	medication group, control group	1. Urine morphine test 2. Electrocardiogram 3. Hamilton Anxiety Scale 4. Withdrawal-symptom evaluation scale	High Risk
Zhuang et al^[13]^	China	2013	75	0	75	20–37 (28.47)	in-patient	Heroin	Yoga, as a mind-body therapy	5 d per week	6 mo	Low	Routine hospital rehabilitation program.	1. Profile of mood states 2. The Medical Outcomes Study 36-item Short-Form Health Survey (SF-36)	Low Risk
Rawson et al^[14]^	USA	2015	135	NA	Education total 66 (28.9% female)/ exercise total 69 (30.40% female)	Education 31.4 (6.5)/ exercise 31.9 (7.4)	NA	methamphetamine	5-min warm- up, 30min of aerobic activity on a treadmill, followed by 15min of weight training and a 5-min cool-down/stretching period	3 times a week	8 wk	structured exercise program: intensity not specified	structured health education protocol	1. Urine drug screen (UDS) 2. Substance Use Inventory (SUI)	Some Concerns
Devi et al^[15]^	India	2014	66	66	0	32.50 ± 9.86	NA	Spasmo proxyvon, Alcohol, Heroin, and Others	Yoga	1 hour and ten minutes of Yoga practices every day	4 wk	intensity not specified	recreational activities	1. Beck Depression Inventory (BDI-II) 2. WHO Quality of Life-BREF	Some Concerns
Trivedi, et al^[16]^	USA	2017	302	181	121	18–65 (39)	in-patient	stimulant	maximum intensity exercise by.walking on incline	3 times per week	12-wk + 24 wk 2 phases of treatment	maximum intensity	Treatment As usual + health education intervention	1. Urine drug screen (UDS) 2. Timeline follow Back (TLFB)	Low Risk
Zhu et al^[17]^	China	2020	100	100	0	32 ± 5 yr (in Experimental group), 30 ± 5 yr (in control group)	NA	heroin, methamphetamine, ketamine, cocaine, ecstasy, Marijuana	Mind-Body Exercise (MBE) including yoga, tai chi or qi gong	60 min a day, five times a week	3 mo	low to moderate	recreational activities	1. QOL-DA questionnaire	Low Risk

*Trial Characteristics.*

### 2.4. Risk of bias assessment

The revised Cochrane Risk of Bias Assessment Tool assessed the risk of bias in the included RCTs (n = 8). The authors assessed the risk of bias independently and in pairs. Based on their judgment, each study was rated as high, low, or some concerns. Disagreements between the reviewers were resolved by discussion or consultation with a third author (MA).

## 3. Results

**Figure 1.**
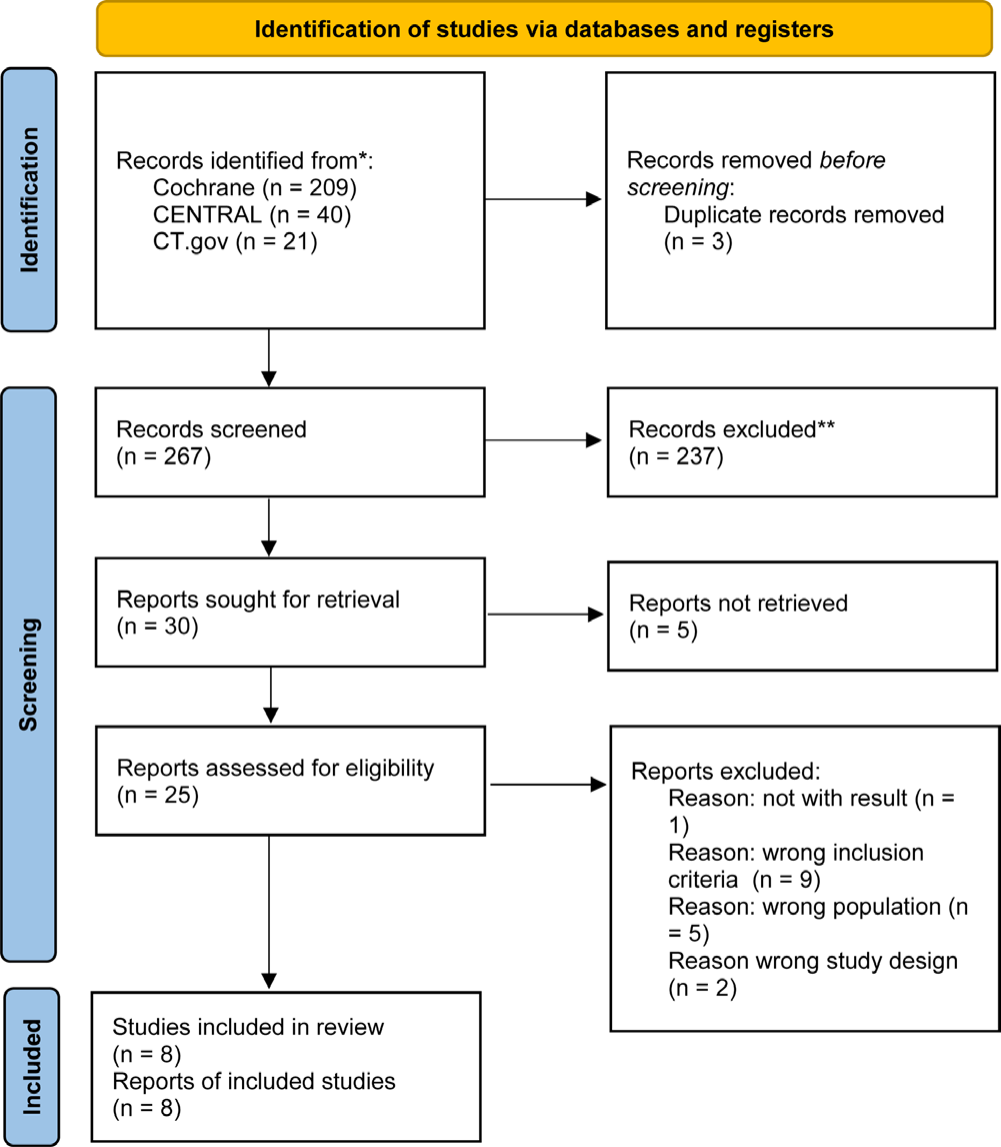
Flowchart illustrating the study procedure following the guidelines outlined in the Preferred Reporting Items for Systematic Reviews and Meta-Analysis (PRISMA). CENTRAL = Cochrane Central Register of Controlled Trial, RCT = randomized controlled trial. The search results were compiled as of 23rd of January 2023. Exclusions were solely conducted by human reviewers.

Initially, the systematic search yielded 267 reports. All these were screened after deduplication. Thirty reports were reviewed for retrieval. After full-text assessment, 17 studies were excluded, while 8 studies were included (Fig. 1).

### 3.1. Trial characteristics

Two of the 8 studies investigated the effect of exercise on alcohol addicts, 2 discussed heroin addicts, one investigated methamphetamine abusers, and 3 analyzed different substance abusers.^[10–17]^ Exercise type-wise, five papers applied yoga with different intensities to their patients.^[11–13,15,17]^ For the other 3, they measure the effect of aerobic exercise on substance use disorder patients.^[10,14,16]^ Four papers assessed QoL, and another 4 discussed craving.^[10,12–17]^ Moreover, 2 measured depression symptoms and 2 studies assessed anxiety levels (Table 1).^[11,12,15]^

### 3.2. Risk of bias assessment

**Figure 2.**
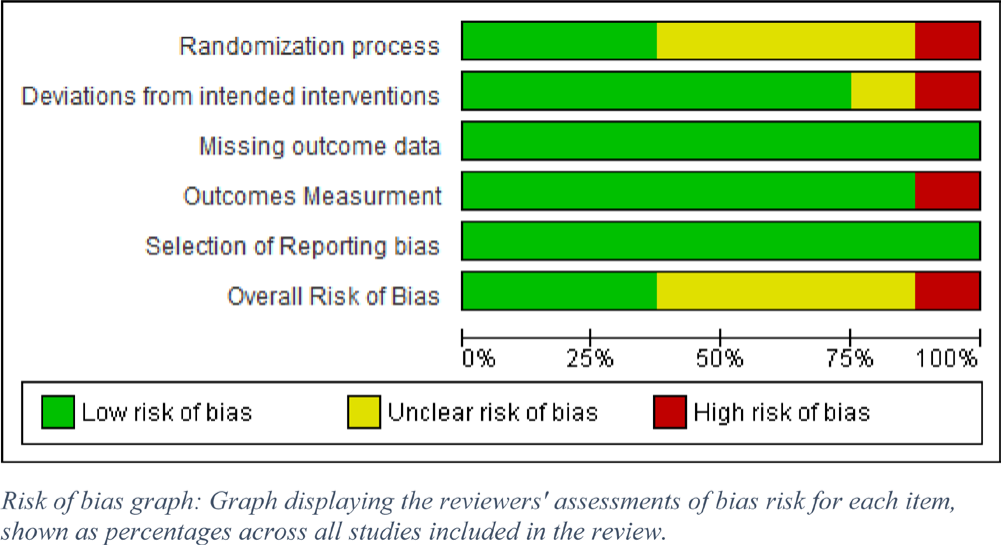
Risk of bias graph: Graph displaying the reviewers’ assessments of bias risk for each item, shown as percentages across all studies included in the review.

**Figure 3.**
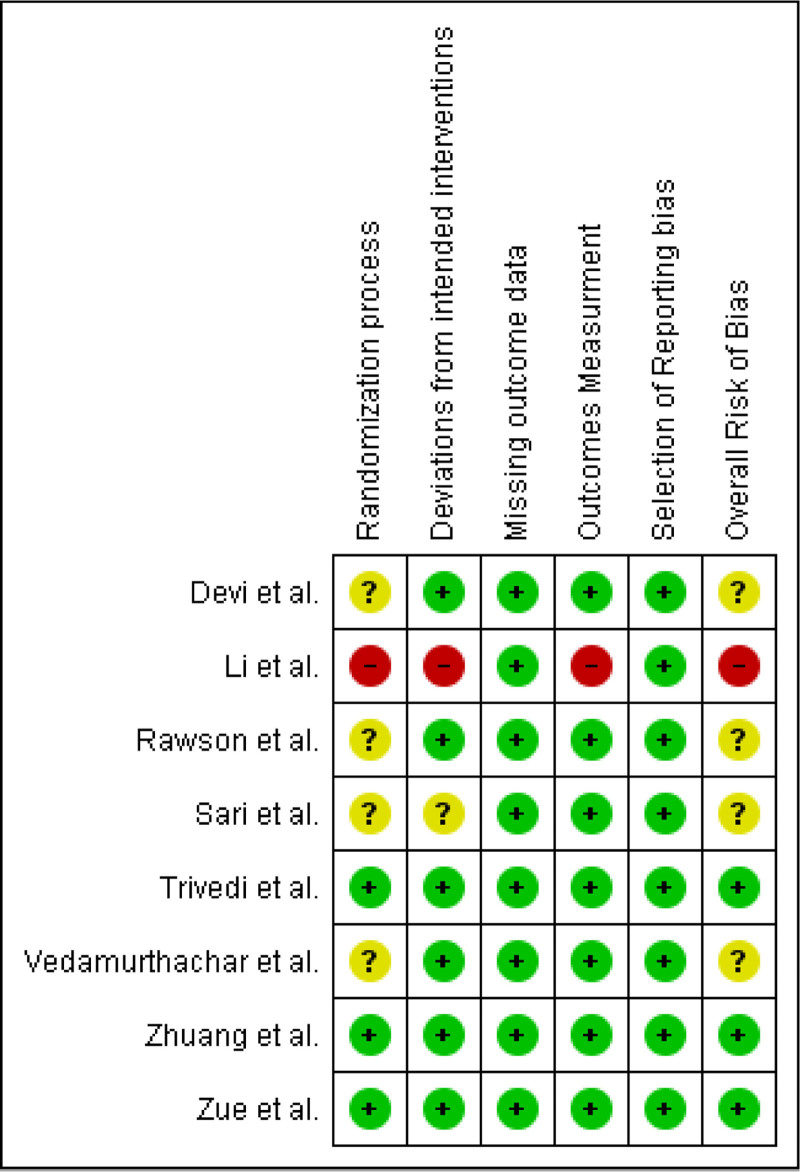
Risk of bias summary.

Three papers were labeled as low risk. Another 4 studies were assessed as having some concerns, while only one study was considered to be at high risk (Figures 2 and 3).

### 3.3. Qualitative synthesis

In relation to the research outcomes, each of the included studies was analyzed from various perspectives. Concerning depression outcome, Vedamurthachar et al aimed to assess the antidepressant effect of Sudarshana Kriya Yoga (SKY) in alcohol-dependent patients. They used the Beck Depression Inventory (BDI) to evaluate depression in participants. Sixty male patients underwent SKY for 2 weeks once daily under the supervision of a yoga therapist. They discovered a decrease in the BDI scores in both arms.^[11]^ Similarly, Devi et al evaluated BDI among users of Spasmo Proxyvon, alcohol, heroin, and other substances. They conducted supervised yoga sessions for 4 weeks, each lasting 1 hour and ten minutes daily, with 66 male drug abusers. Their results were positive, showing a significant decrease in the BDI.^[15]^

Regarding anxiety outcome, both Vedamurthachar et al and Li et al assessed this outcome but used different scales.^[11,12]^ Vedamurthachar et al employed cortisol levels and ACTH, demonstrating a reduction in both arms, with a more pronounced effect in the SKY group.^[11]^ In China, Li et al studied a group of heroin addicts and evaluated the effectiveness of qigong therapy in heroin detoxification. They applied this intervention for 10 days, averaging 2 to 2.5 hours daily, and assessed it using the Hamilton Anxiety Scale. They observed a noticeable reduction in anxiety scores in the qigong treatment group compared with the medication and non-treatment groups.^[12]^

The next outcome was QoL. Sari et al studied alcohol-dependent individuals by assessing physical exercise twice a week for 1 hour over a period of 6 months. The study divided the subjects (n = 117) into 3 groups: treatment as usual, usual treatment in addition to supervised group exercise, and usual treatment in addition to individual physical exercise. Their aim was to assess QoL using the EQ-5D questionnaire and EQ-VAS at 2 time points (before and after treatment). Their results were nonsignificant, except in 1 domain of the EQ-5D questionnaire, where they found no pain or discomfort in the exercise group compared to controls.^[10]^ The other 3 articles on this outcome were included in the meta-analysis. Devi et al found a significant increase in Domains 1, 2, and 3 of the WHOQOL-BREF.^[15]^ Zhuang et al assessed the efficacy of a 6-month yoga intervention among seventy-five female heroin addicts. The yoga group showed an improvement in QoL compared with the control group.^[13]^ Zhe et al assessed QoL by using the QOL-DA questionnaire. Fifty participants underwent mind-body exercises, showing statistically significant improvements in the experimental group.^[17]^

The last outcome, craving frequency or usage, was evaluated in 3 articles. Li et al used a urine morphine test, which showed a noticeable decrease.^[12]^ However, the 2 studies in the meta-analysis showed different results. Trivedi et al studied the effect of absence rates using urine screens in 151 participants assigned to an exercise intervention. The analysis concluded that there was no significant difference between the exercise and control groups.^[16]^ Rawson et al implemented aerobic exercise for 8 weeks among methamphetamine-dependent adults and assessed their outcomes using urine drug screening. Their results showed that these differences were not statistically significant.^[14]^

### 3.4. Meta-analysis

#### 3.4.1. Urine drug screening and physical exercise

**Figure 4.**
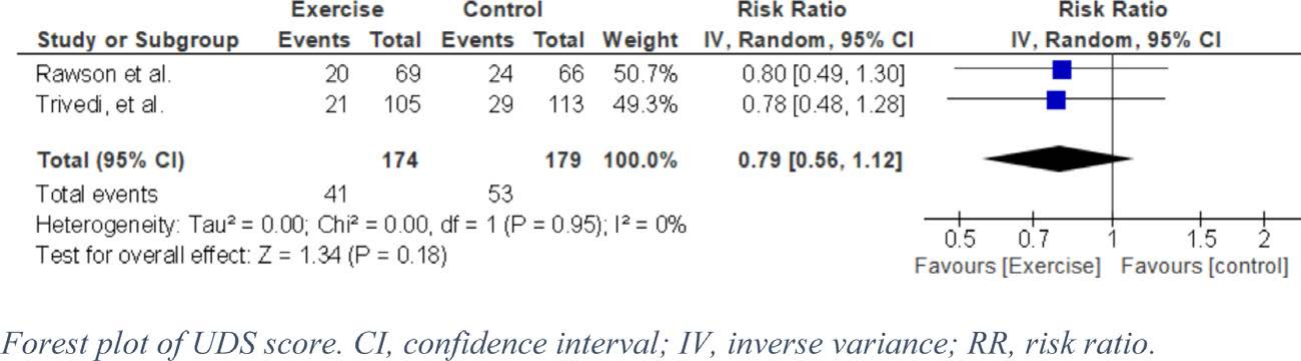
Forest plot of UDS score. CI = confidence interval, IV = inverse variance, RR = risk ratio.

**Figure 5.**
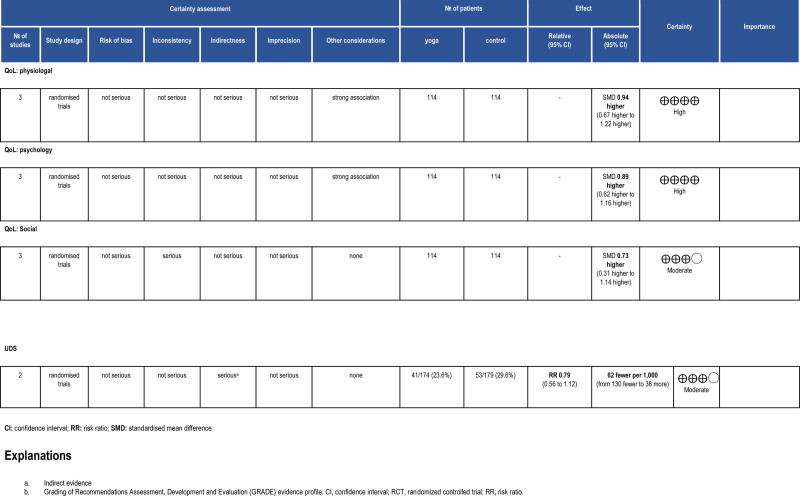
Grading of Recommendations Assessment, Development and Evaluation (GRADE) evidence profile. CI = confidence interval, RCT = randomized controlled trial, RR = risk ratio.

Two RCTs reported the number of participants with positive UDS in both the exercise and control groups with a pooled estimate of 353 participants. The exercise group showed an effect like the control group for this outcome (RR = 0.79, 95% confidence interval [CI]: 0.56–1.12, *P* = .18, I^2^ = 0%) (Fig. 4). Moderate certainty of evidence was estimated (Fig. 5).

#### 3.4.2. Quality of life and exercise: physiology, psychology, and social

**Figure 6.**
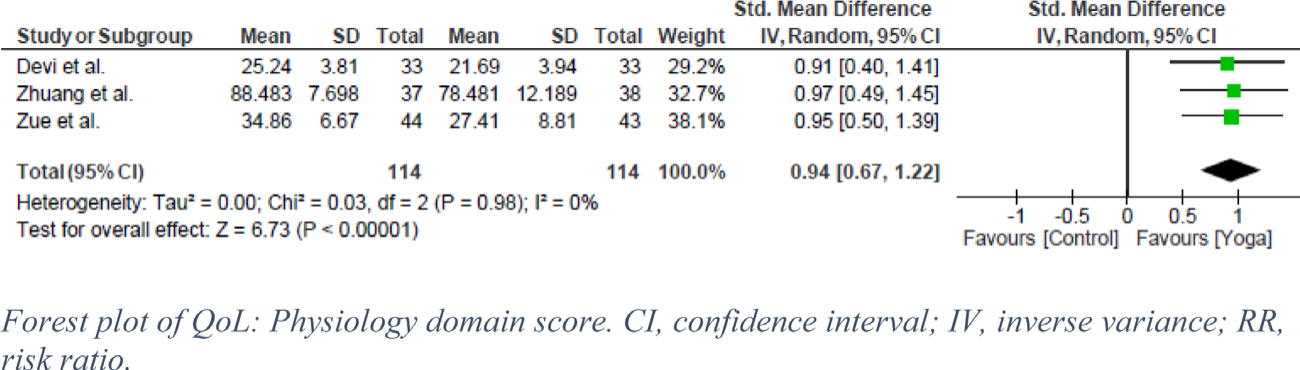
Forest plot of QoL: Physiology domain score. CI = confidence interval, IV = inverse variance, RR = risk ratio.

**Figure 7.**
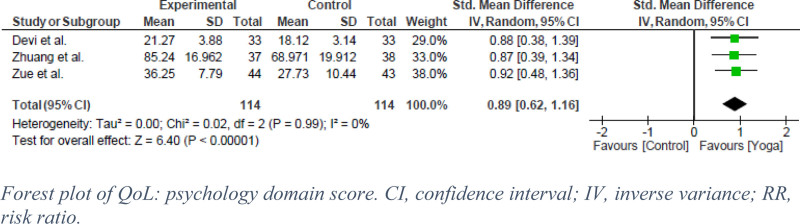
Forest plot of QoL: Psychology domain score. CI = confidence interval, IV = inverse variance, RR = risk ratio.

**Figure 8.**
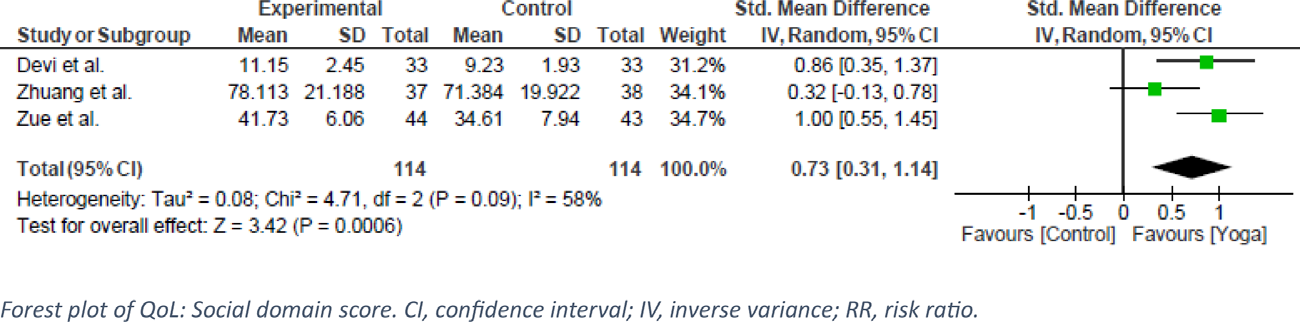
Forest plot of QoL: Social domain score. CI = confidence interval, IV = inverse variance, RR = risk ratio.

Three QoL domains (physiology, psychology, and social) were reported in 3 RCTs that evaluated the effect of yoga in SUD patients, with a pooled analysis of 258 participants. In terms of physiological outcomes, the exercise groups showed significantly greater improvements compared with the control groups receiving standard treatment (SDM = 0.94, 95% CI: 0.67–1.22, *P* < .00001, I^2^ = 0%) (Fig. 6). A high certainty of evidence was found (Fig. 5). Similarly, in the psychology domain, exercise had a statistically significant effect on SUD (SDM = 0.89, 95% CI: 0.62–1.16, *P* < .00001, I^2^ = 0%) (Fig. 7). This is labeled as high certainty of evidence (Fig. 5). Finally, the exercise group showed a significantly superior effect on control in the social domain. However, Zuang et al showed a heterogeneous effect that led to moderate statistical heterogeneity (SDM = 0.73, 95% CI: 0.31–1.14, *P* < .0006, I^2^ = 58%) (Fig. 8).^[13]^ GRADE assessment was estimated to have moderate certainty of evidence (Fig. 5).

## 4. Discussion

This systematic review and meta-analysis evaluated the effectiveness of supervised exercise as an adjunctive therapy for SUD. Our findings suggest that exercise interventions improve quality of life across physical, psychological, and social domains. However, we did not observe significant benefits on craving outcomes. These results highlight exercise as a potentially valuable supportive intervention, though its effects may not extend to all clinical outcomes.

Our results are partly consistent with previous studies. For example, Wang et al^[8]^ reported that exercise was associated with improved abstinence rates and reduced craving, with subgroup analyses suggesting differential effects by exercise type and intensity. In contrast, our analysis did not demonstrate significant effects on craving. Several factors may explain this discrepancy, including the smaller number of eligible trials in our review, variability in outcome measures, and heterogeneity in the populations studied, which included individuals with different substance use profiles and polydrug use. Moreover, our review focused exclusively on randomized controlled trials, which may have limited the pool of available data but ensured methodological rigor.

Nevertheless, our findings add to a growing body of evidence suggesting that exercise can improve mental health outcomes such as depression, anxiety, and overall quality of life in individuals with SUD.^[18,19]^ Exercise may contribute to recovery by reducing stress, enhancing social engagement, and providing a structured, health-promoting routine. Given its low cost, accessibility, and broad health benefits, supervised exercise represents a practical adjunct to conventional treatment programs that often rely primarily on psychotherapy and pharmacotherapy.

Future research should prioritize larger, well-designed randomized trials that stratify by substance type, exercise modality, and follow-up duration. Such studies are needed to clarify the mechanisms underlying the observed benefits and to determine which exercise interventions provide the greatest clinical utility for individuals with SUD.

## 5. Limitations

This review has several limitations. First, some relevant databases such as PsycINFO were not accessible, and only English-language RCTs were included, which may have introduced selection bias. Second, the small number of eligible studies and variability in outcome measures limited the scope of the meta-analysis. Third, the included populations often involved heterogeneous or polydrug users with relatively small sample sizes, which may have reduced the precision of our findings. Finally, although discrepancies between reviewers were resolved through discussion, formal interrater reliability was not calculated. These limitations should be considered when interpreting the results.

## 6. Conclusion

In conclusion, supervised exercise appears to be a promising adjunctive treatment for SUD, with beneficial effects on quality of life and mental health outcomes such as depression and anxiety. However, its impact on craving remains uncertain. Given its safety, accessibility, and low cost, exercise could be feasibly integrated into rehabilitation programs to complement psychotherapy and pharmacotherapy. Future large-scale, high-quality RCTs are needed to clarify the effects of different exercise modalities and to identify which subgroups of patients may benefit most.

## Author contributions

**Conceptualization:** Abdulmajid M. Abdullah.

**Formal analysis:** Anas R. Alserihi, Bader Bashrahil.

**Methodology:** Bader Bashrahil.

**Software:** Anas R. Alserihi.

**Supervision:** Moayyad Alsalem.

**Writing – original draft:** Anas R. Alserihi, Abdulmajid M. Abdullah, Bader Bashrahil, Asim M. Albishry, Khalid W. Alansari, Safwan N. Khan.

**Writing – review & editing:** Anas R. Alserihi, Ahmed Binmahfooz, Ahmad Alsaleh, Majed A. Alharbi, Moayyad Alsalem.

## Supplementary Material


