# Supplementary material for: Supervised exercise as adjunctive treatment for substance use disorder: Systematic review and meta-analysis
Source: Medicine (Baltimore). 2025 Dec 26;104(52):e45370. doi: 10.1097/MD.0000000000045370 (PMC12747012; doi:10.1097/MD.0000000000045370)
Supplement: Supplementary file 1 [file medi-104-e45370-s001.docx]

Search strategy

Cochrane:

Search Name: SUD

Last Saved: 23/01/2023

Comment:

ID Search

#1 MeSH descriptor: [Substance-Related Disorders] explode all trees

#2 MeSH descriptor: [Alcohol-Related Disorders] explode all trees

#3 MeSH descriptor: [Tobacco Use Disorder] explode all trees

#4 MeSH descriptor: [Marijuana Use] explode all trees

#5 MeSH descriptor: [Alcoholism] explode all trees

#6 MeSH descriptor: [Opioid-Related Disorders] explode all trees

#7 (SUD):ti,ab,kw OR (drug addiction):ti,ab,kw OR ("alcohol addiction"):ti,ab,kw OR ("alcohol dependence"):ti,ab,kw OR (alcohol abuse):ti,ab,kw

#8 (cocaine abuse):ti,ab,kw OR (methadone abuse):ti,ab,kw OR (opiate abuse):ti,ab,kw OR (amphetamine abuse):ti,ab,kw

#9 ("Cannabis"):ti,ab,kw OR ("heroine"):ti,ab,kw OR (opioids):ti,ab,kw OR (nicotine):ti,ab,kw OR (smoking):ti,ab,kw

#10 (nicotine):ti,ab,kw OR ("heroine"):ti,ab,kw OR ("Cannabis"):ti,ab,kw

#11 ("poly-substance abuse"):ti,ab,kw OR ("poly-substance abuse"):ti,ab,kw OR ("poly-substance dependence"):ti,ab,kw OR (addiction):ti,ab,kw

#12 #1 OR #2 OR #3 OR #4 OR #5 OR #6 OR #7 #9 OR #10 OR #11

#13 MeSH descriptor: [Exercise] explode all trees

#14 MeSH descriptor: [Exercise Therapy] explode all trees

#15 MeSH descriptor: [Resistance Training] explode all trees

#16 MeSH descriptor: [High-Intensity Interval Training] explode all trees

#17 MeSH descriptor: [Circuit-Based Exercise] explode all trees

#18 MeSH descriptor: [Aquatic Therapy] explode all trees

#19 MeSH descriptor: [Muscle Stretching Exercises] explode all trees

#20 MeSH descriptor: [Walking] explode all trees

#21 MeSH descriptor: [Nordic Walking] explode all trees

#22 MeSH descriptor: [Running] explode all trees

#23 MeSH descriptor: [Circuit-Based Exercise] explode all trees

#24 MeSH descriptor: [Sports] explode all trees

#25 MeSH descriptor: [Soccer] explode all trees

#26 MeSH descriptor: [Football] explode all trees

#27 MeSH descriptor: [Yoga] explode all trees

#28 MeSH descriptor: [Tai Ji] explode all trees

#29 MeSH descriptor: [Qigong] explode all trees

#30 (jogging):ti,ab,kw OR (aerobic stepping):ti,ab,kw OR ("circuit weight training"):ti,ab,kw OR (circuit training):ti,ab,kw OR (circuit):ti,ab,kw

#31 ("aerobic exercise"):ti,ab,kw

#32 ("body-building"):ti,ab,kw OR (sport):ti,ab,kw OR (football):ti,ab,kw OR ("resistance training"):ti,ab,kw OR (yoga):ti,ab,kw

#33 ("Tai Chi"):ti,ab,kw OR ("Tai Chi Chuan"):ti,ab,kw OR ("qigong"):ti,ab,kw OR ("Qi-Gong"):ti,ab,kw OR ("strength training"):ti,ab,kw

#34 #13 OR #14 OR #15 OR #16 OR #17 OR #18 OR #19 OR #20 OR #21 OR #22 OR #23 OR #24 OR #25 OR #26 OR #27 OR #28 OR #29 OR #30 OR #31 OR #32 OR #33

#35 MeSH descriptor: [Randomized Controlled Trial] explode all trees

#36 MeSH descriptor: [Clinical Trial] explode all trees

#37 (RCT):ti,ab,kw OR (randomized controlled trial):ti,ab,kw OR (clinical trial):ti,ab,kw AND (trial):ti,ab,kw

#38 #35 OR #36 OR #37

#39 #12 AND #34 AND #38

Total: 209

Medline search strategy

| Search | Query | Results |
| --- | --- | --- |
| #5 | Search: #1 AND #2 AND #3 AND #4 | 40 |
| #4 | Search:  ((((clinical trials, randomized[MeSH Terms]) OR (controlled clinical trials, randomized[MeSH Terms])) OR (randomized controlled trial[MeSH Terms])) OR (clinical trial[MeSH Terms])) | 379,667 |
| #3 | Search:  **(((((((((((((standard treatment[Title/Abstract]) OR (standard care[Title/Abstract])) OR (usual care[Title/Abstract])) OR (SMART recovery group[Title/Abstract])) OR (treatment as usual[Title/Abstract])) ) OR (health education group[Title/Abstract])) OR (rest[Title/Abstract])) OR (pain education[Title/Abstract])) OR (individual exercise instruction[Title/Abstract])) OR (standard treatment)) OR (standard care)) OR (SMART recovery group)) OR (treatment as usual)** | 1,435,646 |
| #2 | Search:  **((((((((((((((((((((((((((((((activities, physical[MeSH Terms]) OR (activity, physical[MeSH Terms])) OR (jogging[MeSH Terms])) OR (joggings[MeSH Terms])) OR (aerobic exercise[MeSH Terms])) OR (aerobic exercises[MeSH Terms])) OR (football[MeSH Terms])) OR (footballs[MeSH Terms])) OR (resistance training[MeSH Terms])) OR (yoga[MeSH Terms])) OR (tai chi[MeSH Terms])) OR (qigong[MeSH Terms])) OR (recreation therapy[MeSH Terms])) OR (exercise intervention)) OR (exercise in addition to standard treatment)) OR (exercise as adjunct intervention)) OR (excercise)) OR (running)) OR (walking)) OR (jogging)) OR (physical activity)) OR (body building)) OR (resistance training)) OR (aerobic stepping)) OR (exercise)) OR (group excercise)) OR (exercise bike)) OR (high intensity interval training)) OR (circuit training)) OR (aerobic exercise)) OR (ball games)** | 843,933 |
| #1 | Search:  **((((((((((((((((((((((((((((((((((((((((((abuse, substance[MeSH Terms]) OR (abuses, substance[MeSH Terms])) OR (addiction, substance[MeSH Terms])) OR (alcohol, drug abuse, and mental health administration[MeSH Terms])) OR (abuse drugs[MeSH Terms])) OR (alcohol abuse[MeSH Terms])) OR (abuse, cocaine[MeSH Terms])) OR (addiction, cocaine[MeSH Terms])) OR (cocaine abuse[MeSH Terms])) OR (cocaine addiction[MeSH Terms])) OR (addiction, opiate[MeSH Terms])) OR (dependence, opiate[MeSH Terms])) OR (opiate addiction[MeSH Terms])) OR (abuse, cannabis[MeSH Terms])) OR (cannabis abuse[MeSH Terms])) OR (cannabis dependence[MeSH Terms])) OR (cannabis abuse[MeSH Terms])) OR (abuse, amphetamine[MeSH Terms])) OR (addiction, amphetamine[MeSH Terms])) OR (amphetamine abuse[MeSH Terms])) OR (amphetamine addiction[MeSH Terms])) OR (abuse, heroin[MeSH Terms])) OR (addiction, heroin[MeSH Terms])) OR (dependence, heroin[MeSH Terms])) OR (disorder, opioid related[MeSH Terms])) OR (cannabis smoking[MeSH Terms])) OR (dependence, nicotine[MeSH Terms])) OR (disorder, nicotine use[MeSH Terms])) OR (substance use disorder[MeSH Terms])) OR (substance use disorders[MeSH Terms])) OR (substance use disorder[Title/Abstract])) OR (cocaine abuse[Title/Abstract])) OR (alcohol abuse[Title/Abstract])) OR (addiction[Title/Abstract])) OR (poly substance[Title/Abstract])) OR (poly-substance[Title/Abstract])) OR (methadone abuse[Title/Abstract])) OR (heroin abuse[Title/Abstract])) OR (drug abuse[Title/Abstract])) OR (SUD[Title/Abstract])) OR (patient seeking treatment for SUD[Title/Abstract])) OR (patient treating for substance use[Title/Abstract])) OR (addictive patients[Title/Abstract])** | 362,131 |

Total: 40

CT.gov search strategy

Condition: Substance Use Disorders OR alcohol abuse OR cocaine abuse OR Cannabis OR heroine OR amphetamine abuse OR Nicotine OR Heroine OR Poly-substance abuse

Other terms: Exercise OR High Intensity Interval Training OR Resistance Training OR Aquatic Therapy OR Aerobic exercise

Search Limits: Study Type [interventional] Study results [with results]

Total: 21
